# Thermoelectric properties of M_2_BS_2_ (M = Ti, Zr, Hf) monolayers: An Ab initio study

**DOI:** 10.1371/journal.pone.0339290

**Published:** 2026-01-21

**Authors:** Shengzhao Wang, Lanli Chen, Jinfan Song

**Affiliations:** 1 Nanyang Institute of Technology, School of Mathematics and Physics, Nanyang, Henan, China; 2 Henan Province Engineering Technology Research Center of New Optoelectronic and Storage Materials, Nanyang, Henan, China; National Research Centre, EGYPT

## Abstract

The thermoelectric properties of two-dimensional M_2_BS_2_ (M = Ti, Zr, Hf) materials were investigated in this study by using first-principles calculations. The phonon dispersion indicate that the high-frequency branches are dominated by B vibrations, while the mid- and low-frequency branches are primarily influenced by M and S atoms. All three compounds are confirmed to be dynamically stable. The lattice thermal conductivity is primarily contributed by acoustic and low-frequency optical phonons, with its overall magnitude determined by relaxation times, group velocities, and Grüneisen parameters. The resulting thermal conductivities follow the sequence *k*_*l*_(Ti_2_BS_2_) > *k*_*l*_(Hf_2_BS_2_) > *k*_*l*_(Zr_2_BS_2_), reaching 2.35 W·m^-1^·K^-1^, 2.14 W·m^-1^·K^-1^, and 2.05 W·m^-1^·K^-1^ at room temperature, respectively. Monolayer Hf_2_BS_2_ maintains relatively high Seebeck coefficients and power factors under either doping polarity, achieving a peak thermoelectric figure of merit of 1.74 in the n-type configuration. These findings provide a strong theoretical foundation for designing novel, high-performance thermoelectric device materials.

## 1. Introduction

Driven by increasing energy demands and growing environmental concerns, the development of high-efficiency, low-cost, and eco-friendly thermoelectric materials has become a critical research frontier [1,2]. The direct conversion of heat to electricity makes thermoelectric materials essential for waste heat recovery and sustainable energy utilization. Traditional thermoelectric materials, however, suffer from high costs and limited efficiency, prompting researchers to explore novel material systems [3]. Two-dimensional materials—such as graphene, MXenes, and their derivatives—are emerging as promising candidates because their unique electronic structures and physical properties enable exceptional thermoelectric performance [4].

Superior carrier mobility, inherently low lattice thermal conductivity, and highly tunable electronic structures make two-dimensional (2D) materials promising candidates for next-generation thermoelectric applications [5–7]. Their exceptional mechanical flexibility further makes them suitable for wearable and flexible thermoelectric devices [8,9]. Among the rapidly expanding family of 2D materials, MXenes—transition-metal carbides, nitrides, or carbonitrides—stand out due to their distinctive layered architectures, metallic-to-semiconducting electronic properties, high electrical conductivity, and robust mechanical strength [10]. Surface functionalization or intercalation of MBenes can produce derivative phases whose structures and properties differ significantly from those of the parent compounds, often resulting in substantially enhanced thermoelectric performance. First-principles investigations of MBene-derived systems, such as M_2_BS_2_ (M = Ti, Zr, Hf), can quantitatively reveal their thermoelectric potential, elucidate the underlying electron and phonon transport mechanisms, and guide targeted optimization strategies. These atomistic insights are crucial for accelerating the discovery and deployment of novel high-performance thermoelectric materials, underscoring the practical significance of research on 2D thermoelectrics within the broader context of sustainable energy technologies [11–14].

Thermoelectric applications of 2D materials have advanced significantly in recent years. First-principles calculations have identified several 2D candidates—including γ-graphyne, MXenes, black phosphorus, and SnTe—with promising thermoelectric performance [15–17]. Experimentally, prototype 2D thermoelectric devices have been successfully fabricated, demonstrating encouraging conversion efficiencies [18]. Despite these advances, significant challenges remain. The figure of merit for most 2D thermoelectrics remains too low for practical deployment [19]. Future efforts must therefore focus on discovering novel 2D compounds and developing innovative modification strategies, including surface functionalization, strain engineering, and heterostructure design. Only by simultaneously enhancing performance and stability while reducing fabrication costs can 2D thermoelectrics transition from laboratory curiosities to widespread energy-harvesting technologies [20]. In particular, we compare the thermoelectric properties of the Hf_2_BS_2_ monolayer with those of several representative two-dimensional thermoelectric materials—namely, SnSe monolayer, MoS_2_, and Bi_2_Te_3_ thin films—that have been extensively studied in the literature. Theoretical studies predict that SnSe monolayers can achieve a high ZT of ~3.0 at 900 K, but no reliable experimental ZT has been reported for a true monolayer, with the best experimental result being only 0.12 for nanosheets at 310 K [21–26]. MoS_2_ monolayers exhibit a theoretical ZT of 0.58 at room temperature, which can increase to 2.79 under high pressure (25–50 GPa) at 700 K; experimentally, the highest ZT achieved is 0.6 using an organic superlattice at 373 K [27–31]. Bi_2_Te_3_ thin films, a commercial thermoelectric standard, have theoretical ZT values of 1.5–2.3 under quantum confinement, with the best experimental values being 1.28 for alloyed films and 2.4 for superlattices [32–35]. In contrast, the Hf_2_BS_2_ monolayer investigated in this work demonstrates a ZT of 1.74 at 300 K under ambient pressure, which is higher than the ambient-condition theoretical value of MoS_2_ and comparable to the best experimental results of Bi_2_Te_3_ thin films. Importantly, this performance is achieved without the need for high temperature, high pressure, or complex interface engineering, underscoring the practical potential of Hf_2_BS_2_ for thermoelectric applications.

In conclusion, conducting a first-principles investigation of the thermoelectric properties of two-dimensional M_2_BS_2_ materials aims to reveal—through rigorous theoretical calculations—their potential for efficient heat-to-electricity conversion and to elucidate the underlying electron and phonon transport mechanisms [36,37]. Beyond clarifying intrinsic performance limits and transport physics, this study will provide actionable guidance for material optimization and device engineering, accelerate the development of next-generation thermoelectrics, and ultimately contribute to addressing global energy and environmental challenges.

## 2. Computational details

All calculations in this study are based on density functional theory (DFT) and were performed using the Vienna Ab-initio Simulation Package (VASP). The exchange–correlation energy was treated within the generalized gradient approximation (GGA) using the Perdew–Burke–Ernzerhof (PBE) functional [38–41]. A 25 Å vacuum slab was inserted perpendicular to the layers to eliminate spurious interlayer interactions, and Van der waals forces were accounted for using the vdW-DF2 functional [42]. Electronic band structures were subsequently refined with the Heyd–Scuseria–Ernzerhof (HSE06) hybrid functional to obtain accurate band gaps and dispersion relations [43].

Electrical transport coefficients were calculated using the BoltzTraP code by solving the Boltzmann transport equation within the constant relaxation time approximation on a dense 45 × 45 × 1 k-point mesh [44]. A plane-wave cutoff energy of 600 eV was employed to ensure numerical accuracy, while total energy and force convergence criteria were tightened to 1 × 10^−8^ eV and 1 × 10^−7^ eV/Å, respectively. Structural relaxations and ground-state calculations utilized an 11 × 11 × 1 Γ-centered k-point grid [45,46]. Lattice dynamical and thermal properties were subsequently computed by interfacing VASP with ShengBTE, enabling fully first-principles evaluation of phonon dispersions, phonon lifetimes, and lattice thermal conductivity. Anharmonic interatomic force constants (IFCs) were calculated using 4 × 4 × 1 supercells generated with Phonopy. Third-order IFC matrices were obtained with Thirdorder by retaining interactions up to the 13th nearest neighbors, ensuring convergence. The phonon Boltzmann transport equation was subsequently solved with ShengBTE to yield lattice thermal conductivity and related thermal-transport data; a 50 × 50 × 1 q-point mesh was adopted in all ShengBTE runs [47,48].

## 3. Results and discussion

### 3.1. Crystal structure and phonon dispersion

**Fig 1:**
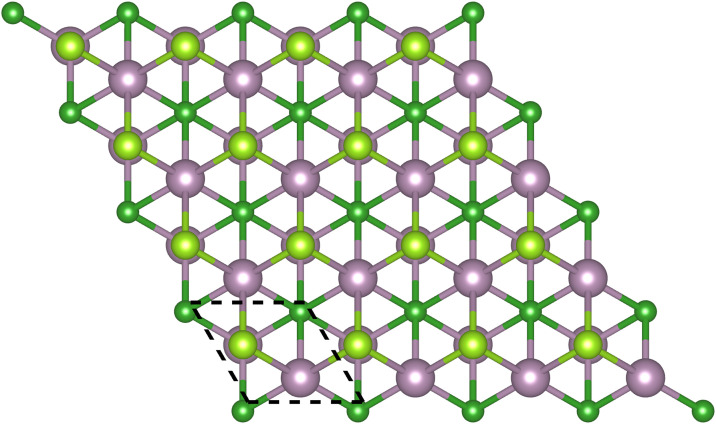
Top view of M_2_BS_2_ structure.

**Fig 2:**
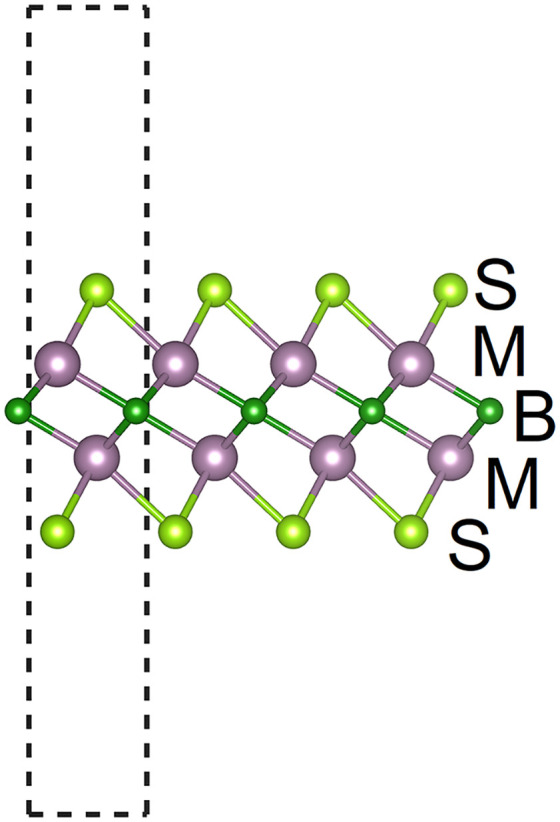
Side view of M_2_BS_2_ structure.

As shown in Fig 1 and Fig 2, M_2_BS_2_ is a ternary layered boride composed of transition-metal atoms (M), boron, and sulfur. It is synthesized by selectively etching a parent MAB-phase ceramic, followed by surface termination with sulfur, resulting in an MXene derivative. The compound crystallizes in the trigonal space group $\text{p}\overset{―}{\text{3}}\text{m1}$ (No. 164). Because Ti, Zr, and Hf differ in atomic radius and electronegativity, the in-plane lattice parameters a (= b) increase monotonically with the size of the M atom, while the out-of-plane lattice parameter c remains nearly constant due to the two-dimensional nature of the layers. The overall M_2_BS_2_ structural stability exhibits an M-dependent trend that parallels the behavior observed in S-terminated MXenes, providing a reliable structural foundation for further applications.

**Fig 3:**
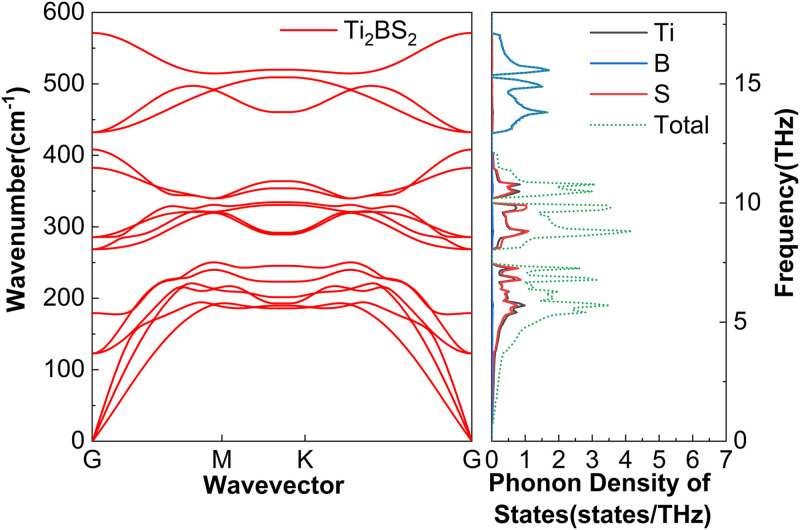
Phonon dispersion and the corresponding projected phonon density of states of Ti_2_BS_2_.

The phonon dispersion of M_2_BS_2_ are determined by its layered structure, atomic masses, and bond strengths. Each primitive cell contains five atoms, resulting in fifteen phonon branches: three acoustic and twelve optical. Fig 3–Fig 5 show that the high-frequency region is dominated by boron vibrations. Because boron is light and forms strong covalent bonds, the associated optical branches exhibit obvious dispersion. The mid-frequency region is influenced by sulfur and the transition metal M, whose intermediate masses and bond strengths produce moderate dispersion. The low-frequency region includes the acoustic branches and low-lying optical modes, both dominated by the heavier M and S atoms. The projected phonon density of states (PhDOS) supports this assignment: sharp boron-derived peaks appear at high frequencies, corresponding to the upper optical branches; sulfur and metal peaks dominate the mid-frequency range, attributable to the lower optical branches; and significant metal- and sulfur-derived contributions at low frequencies are associated with the acoustic modes. No imaginary frequencies are observed for any of the M_2_BS_2_ compounds, confirming their dynamical stability.

**Fig 4:**
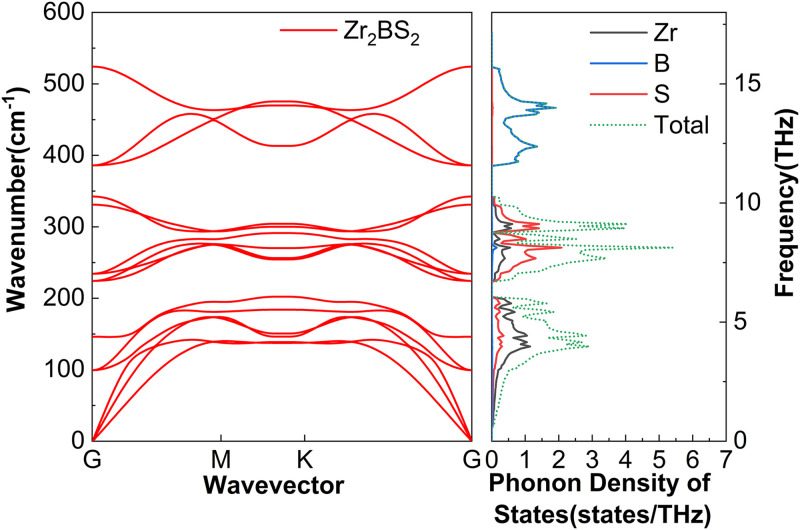
Phonon dispersion and the corresponding projected phonon density of states of Zr_2_BS_2_.

**Fig 5:**
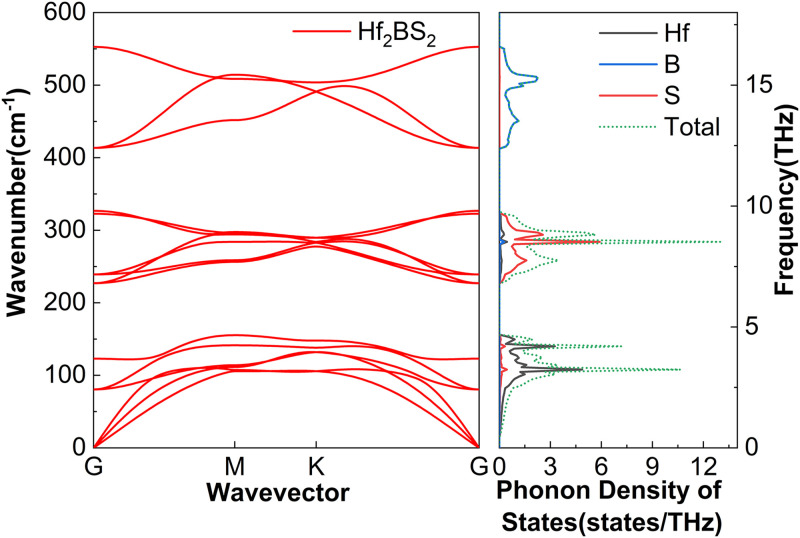
Phonon dispersion and the corresponding projected phonon density of states of Hf_2_BS_2._

Because the high-frequency (B-dominated) modes are strongly scattered, they contribute little to the lattice thermal conductivity. By contrast, the medium and low frequencies primarily correspond to the vibrations of M and S atoms, which contribute more significantly to thermal conductivity.

### 3.2. Phonon transport properties

Generally, the lattice thermal conductivity $\kappa_{l}$ of a material is positively correlated with the phonon relaxation time (the inverse of the phonon scattering rate) and the phonon group velocity. Conversely, it is negatively correlated with the Grüneisen parameter (*γ*), the phase-space volume, the number of scattering channels, and the phonon scattering rate itself. Using the ShengBTE package, the exact lattice thermal conductivity $\kappa_{l}$ can be determined. The lattice thermal conductivity is calculated as follows [49]:


$$\kappa_{l}\;=\frac{\text{1}}{NVk_{B}T^{\text{2}}}\sum_{\lambda }f_{\text{0}}{\left(f_{\text{0}}+\text{1}\mathrm{\backslash rightleft}(\hbar \omega_{\lambda }\right)}^{\text{2}}{\left(v_{\lambda }^{z}\right)}^{\text{2}}\tau_{\lambda }$$ (1)

Here,$\;v_{\lambda }$ is the phonon group velocity, $\tau_{\lambda }$ is the phonon relaxation time (the reciprocal of the scattering rate), and $\frac{\text{1}}{\tau_{\lambda }}$ is determined by two-phonon scattering due to isotope disorder and three-phonon anharmonic scattering. Therefore, the phonon relaxation time $\tau_{\lambda }$, the anharmonic three-phonon scattering rate $\frac{\text{1}}{\tau_{\lambda }}$, the phonon group velocity $v_{\lambda }$, and the Grüneisen parameter *γ* together become the dominant factors affecting the lattice thermal conductivity [50,51].

#### 3.2.1. Phonon relaxation time.

The phonon lifetime, *τ*, a key parameter governing lattice thermal conductivity, is obtained from ShengBTE as the reciprocal of the total scattering rate. This total scattering rate includes contributions from isotopic scattering, anharmonic three-phonon processes, and other mechanisms. Although high-frequency optical modes in M_2_BS_2_ contribute to heat transport, the lattice thermal conductivity is predominantly governed by the acoustic branches and low-lying optical modes.

**Fig 6:**
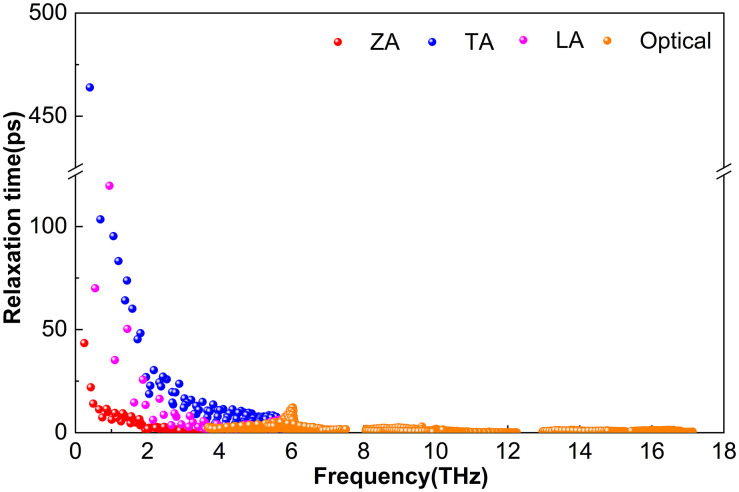
Phonon Relaxation Time of Ti_2_BS_2._

As shown in Fig 6, Ti_2_BS_2_ exhibits significantly longer phonon lifetimes for acoustic branches compared to optical branches. The in-plane transverse acoustic (TA) vibrations persist the longest, reaching 463.9 ps, followed by longitudinal acoustic (LA) modes with lifetimes below 119.8 ps. In contrast, out-of-plane flexural acoustic (ZA) modes have the shortest acoustic lifetimes, under 43.4 ps. Within the 3.7–17.3 THz range, optical phonons dominate the lifetime spectrum, all remaining below 12.2 ps. Between 3.7 and 6.5 THz, the lifetimes of these optical phonons overlap with those of the ZA, TA, and LA branches. At 300 K, all four branches exhibit appreciable lifetimes. The reduced phonon–phonon scattering at low frequencies results in extended lifetimes, allowing acoustic phonons to maintain coherence over long transport distances and thereby contributing to Ti_2_BS_2_’s relatively high lattice thermal conductivity. Conversely, optical phonons in the mid- to high-frequency range (4–17 THz) have much shorter lifetimes, mostly between 0 and 12.2 ps.

**Fig 7:**
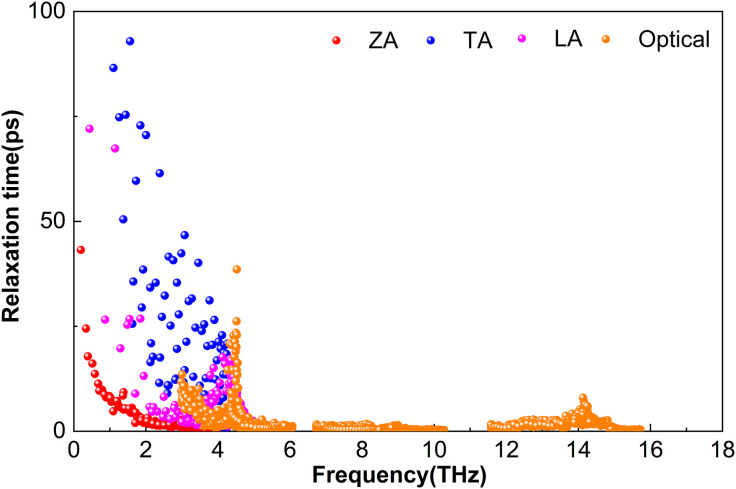
Phonon Relaxation Time of Zr_2_BS_2._

Fig 7 shows that Zr_2_BS_2_ exhibits relatively short phonon lifetimes across both acoustic and optical branches. The TA phonons have the longest lifetimes, yet remain below 92.9 ps; the LA modes reach up to 72.0 ps, while the ZA branch is limited to approximately 43.2 ps. In the 3.0–15.7 THz range, phonon lifetimes are dominated by optical phonons, all below 38.6 ps. Between 3.2 and 5.2 THz, these optical phonon lifetimes overlap with those of the ZA, TA, and LA branches. At 300 K, all phonon branches in Zr_2_BS_2_ exhibit significantly shorter lifetimes than their counterparts in Ti_2_BS_2_. Despite weak low-frequency phonon–phonon scattering, the shorter absolute lifetimes reduce the lattice thermal conductivity of Zr_2_BS_2_. Mid- to high-frequency optical phonons (6.7–15.7 THz) exhibit brief lifetimes, predominantly ranging from 0 to 14.1 ps.

**Fig 8:**
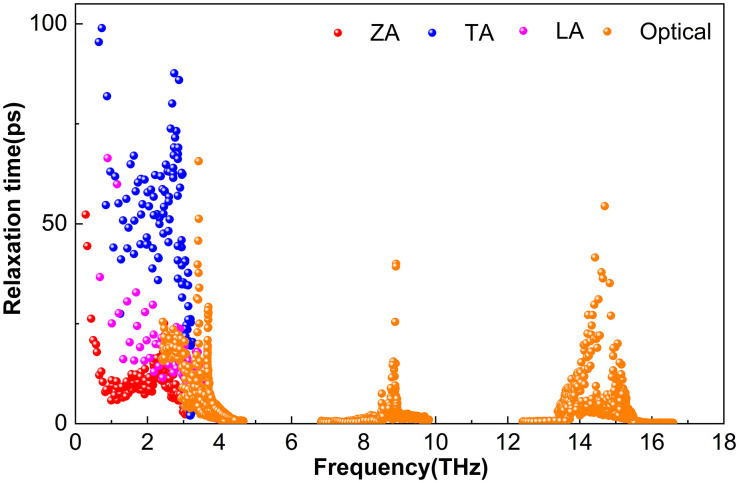
Phonon Relaxation Time of Hf_2_BS_2._

Fig 8 shows that Hf_2_BS_2_ exhibits relatively short phonon lifetimes across both acoustic and optical branches. The TA modes have the longest lifetimes, though they remain below 99.0 ps; the LA branch reaches up to 66.4 ps, while the ZA branch is limited to approximately 52.3 ps. Within the 2.5–16.6 THz range, phonon lifetimes are primarily governed by optical phonons, all of which are below 65.7 ps. Between 2.5 and 3.5 THz, these optical phonon lifetimes overlap with those of the ZA, TA, and LA branches. At 300 K, all phonon branches in Hf_2_BS_2_ exhibit relatively short lifetimes. Despite weak low-frequency phonon–phonon scattering, the absolute phonon lifetimes in Hf_2_BS_2_ remain shorter than those in Ti_2_BS_2_, resulting in a lattice thermal conductivity that is lower than Ti_2_BS_2_ but slightly higher than Zr_2_BS_2_. In the mid- to high-frequency range (6.8–16.6 THz), optical phonon lifetimes are short, predominantly ranging from 0 to 54.4 ps.

#### 3.2.2. Phonon group velocity.

**Fig 9:**
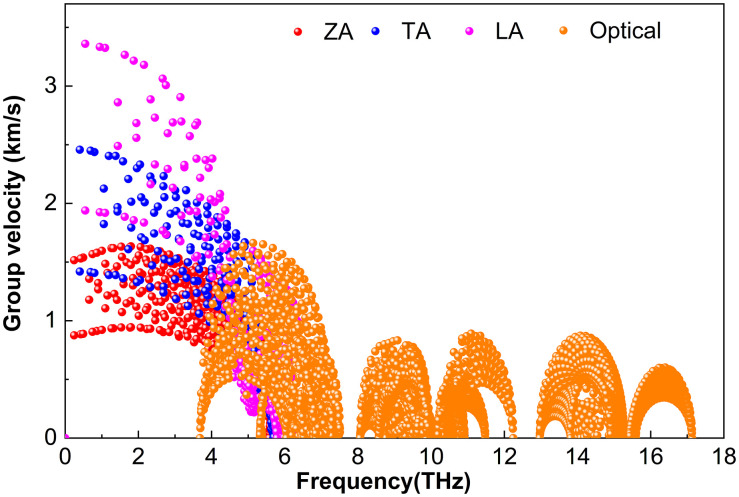
Phonon group velocity of Ti_2_BS_2._

Phonon group velocity***υ***—the derivative of frequency with respect to the wave vector—governs the efficiency of heat transport. Fig 9–Fig 11 show ***υ*** for M_2_BS_2_ at 300 K. Although the atomic masses of Ti, Zr, and Hf increase progressively, the heavier M atoms only slightly reduce the overall sound velocities (since heavier masses slow lattice vibrations). However, the strong scattering caused by the light B and intermediate-mass S atoms largely masks this mass effect. Consequently, the phonon group velocities of Ti_2_BS_2_, Zr_2_BS_2_, and Hf_2_BS_2_ remain nearly identical. The nearly equal group velocities, combined with similar anharmonic scattering strengths discussed earlier, result in comparable and intrinsically low lattice thermal conductivities for all three compounds.

**Fig 10:**
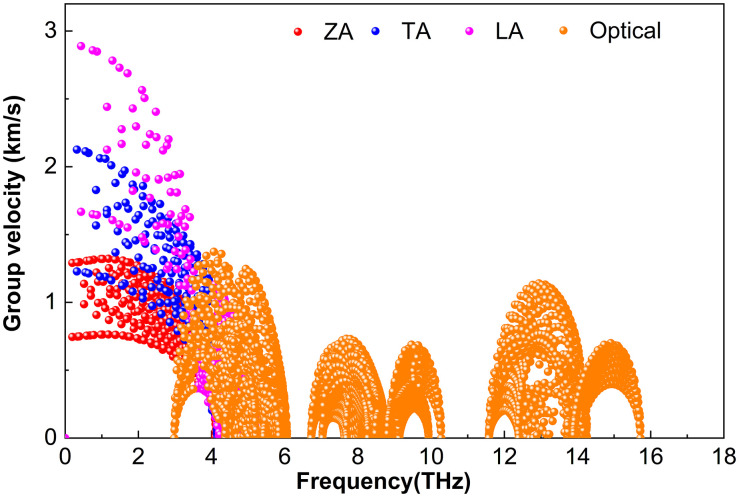
Phonon group velocity of Zr_2_BS_2._

**Fig 11:**
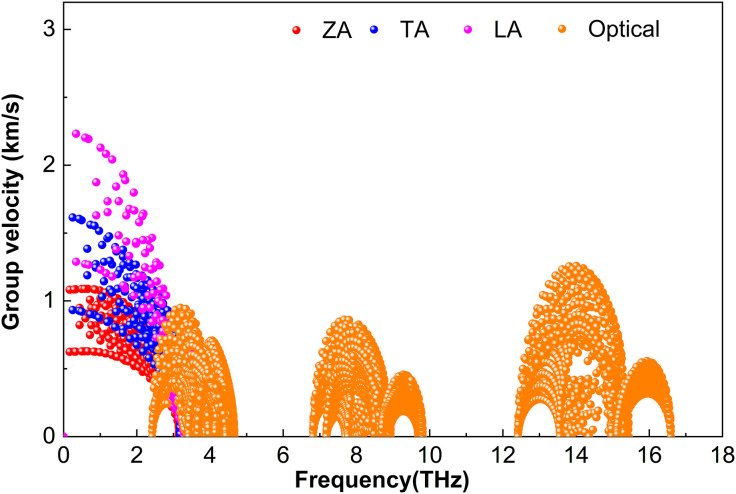
Phonon group velocity of Hf_2_BS_2._

Fig 9 shows that in Ti_2_BS_2_, the acoustic branches (ZA, TA, LA) exhibit significantly higher group velocities than the optical branches. All acoustic modes lie below approximately 6.5 THz. Among these, the flexural ZA branch has the lowest velocities, peaking at about 1.63 km ∙ s^-1^. The LA branch reaches the highest velocities, with some modes attaining approximately 3.4 km ∙ s^-1^, while certain TA modes approach around 2.5 km ∙ s^-1^. Optical phonons in the 3.7–7.5 THz range reach velocities of up to approximately 1.7 km/s, whereas those in the high-frequency optical region (8.1–17.1 THz) are significantly slower, with a maximum velocity of only about 0.9 km/s.

Fig 10 shows that the phonon group velocity of Zr_2_BS_2_ closely resembles that of Ti_2_BS_2_ in Fig 9. The acoustic branches (ZA, TA, LA) exhibit higher velocities than the optical branches and are confined below approximately 5.1 THz. Among the acoustic branches, the flexural ZA branch is the slowest, peaking at about 1.3 km/s, whereas the LA branch attains the highest velocities, with a small fraction of modes reaching approximately 2.9 km/s. A subset of TA modes approaches around 2.1 km/s. Optical phonons between 3.0 and 6.1 THz reach velocities up to approximately 1.4 km/s, while those in the high-frequency optical region (6.7–15.7 THz) are significantly slower, with a maximum velocity of only about 1.1 km/s.

Fig 11 presents the phonon group velocity of Hf_2_BS_2_. The acoustic branches (ZA, TA, LA) exhibit higher velocities than the optical branches and are confined to approximately 4.0 THz. Among the acoustic branches, the flexural ZA branch is the slowest, peaking at about 1.1 km ∙ s^-1^. The LA branch shows the highest velocities, with a small fraction of modes reaching approximately 2.2 km ∙ s^-1^, while some TA modes approach around 1.6 km ∙ s^-1^. Optical phonons between 2.4 and 4.7 THz reach velocities up to approximately 0.9 km ∙ s^-1^, whereas those in the high-frequency optical region (6.8–16.6 THz) exhibit modest velocities, with a maximum of only about 1.3 km ∙ s^-1^.

#### 3.2.3. Grüneisen parameter.

**Fig 12:**
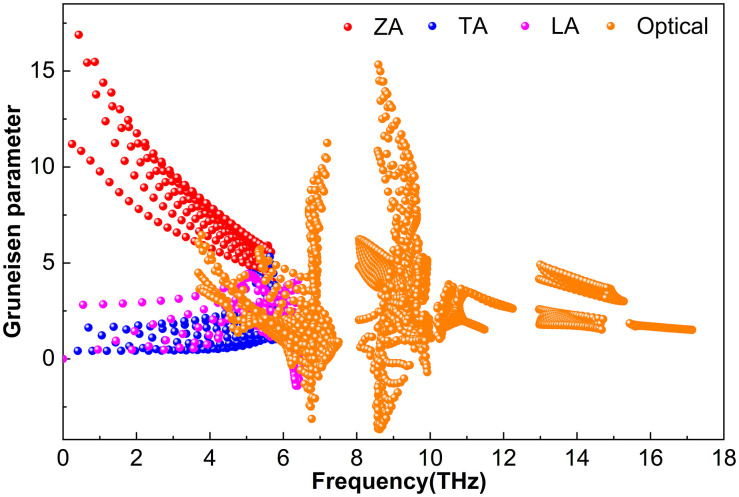
Grüneisen parameter of Ti_2_BS_2._

Fig 12–Fig 14 display the *γ* parameters of various phonon modes in the M_2_BS_2_ materials. While the overall patterns are similar, the magnitudes vary. Fig 12 shows that for Ti_2_BS_2_, both acoustic and optical phonons have relatively small *|γ|* values. Near 0.43 THz, the absolute value of *γ* for the ZA branch reaches approximately 16.9. In the TA and LA branches, *|γ|* is significantly smaller, peaking at 5.3 and 1.4, respectively. Within the 3.7–17.1 THz range, the optical modes exhibit *|γ|* values that generally do not exceed about 15.3.

**Fig 13:**
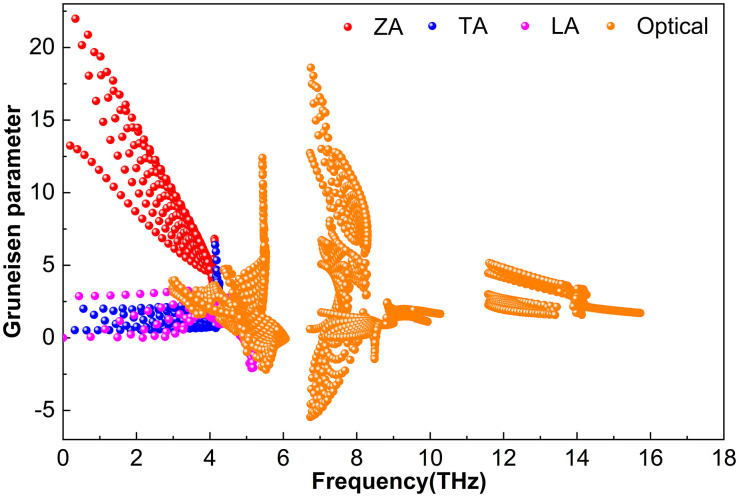
Grüneisen parameter of Zr_2_BS_2._

**Fig 14:**
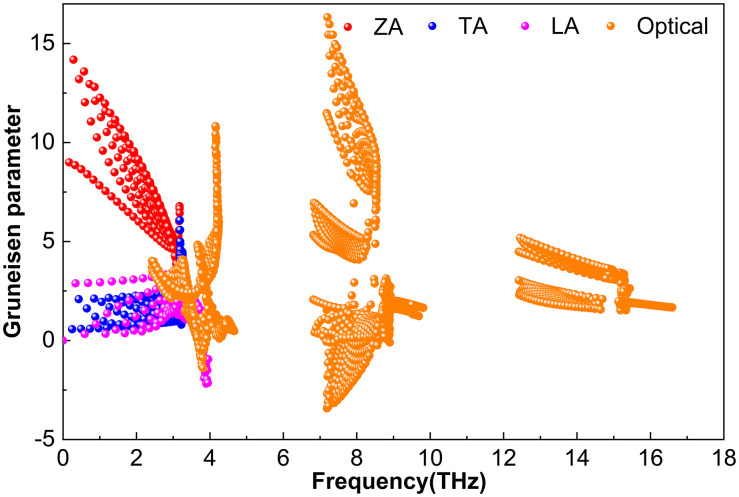
Grüneisen parameter of Hf_2_BS_2._

Fig 13 displays the mode-resolved Grüneisen parameters (γ) for Hf_2_BS_2_, revealing relatively large magnitudes in both acoustic and optical phonon modes. Near 0.3 THz, the ZA mode reaches *|γ|* ≈ 22.0. In contrast, the TA and LA modes show much smaller *|γ|* values, peaking at 6.4 and 2.1, respectively. Within the 3.0–15.7 THz range, the optical modes have *|γ|* values that do not exceed approximately 18.6.

Fig 14 shows the mode-resolved Grüneisen parameters (γ) for Hf_2_BS_2_, revealing relatively small magnitudes for both acoustic and optical phonon modes. Near 0.3 THz, the ZA branch reaches *|γ|* ≈ 14.2. In the TA and LA branches, *|γ|* remains modest, peaking at 3.2 and 2.1, respectively. Over the 2.4–16.6 THz range, the optical modes show *|γ|* values that generally do not exceed approximately 16.3.

These results reveal that the absolute value of *γ* for Zr_2_BS_2_ is slightly larger than those for Ti_2_BS_2_ and Hf_2_BS_2_, indicating stronger anharmonicity and more pronounced phonon–phonon interactions in this crystal. Consequently, phonons are scattered more frequently during propagation, reducing the efficiency of heat transport and resulting in a somewhat lower lattice thermal conductivity. When combined with the effects of relaxation time, phonon group velocity, and the Grüneisen parameter, this ultimately leads to the relationship *k*_*l*_(Ti_2_BS_2_) > *k*_*l*_(Hf_2_BS_2_) > *k*_*l*_(Zr_2_BS_2_). Additionally, the presence of negative *γ* values in all M_2_BS_2_ compounds suggests the possibility of negative thermal expansion.

#### 3.2.4. Lattice thermal conductivity.

**Fig 15:**
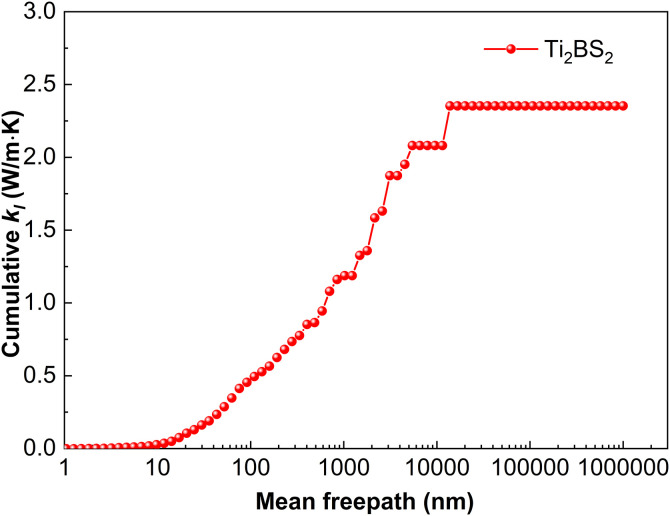
Cumulative lattice thermal conductivity of Ti_2_BS_2._

Fig 15 shows the cumulative lattice thermal conductivity *k*_*l*_ of Ti_2_BS_2_ at 300 K as a function of the phonon mean free path (MFP). Sample size is a critical factor: *k*_*l*_ stops increasing once the MFP exceeds 13,848.9 nm, indicating that all heat-carrying phonons have MFPs below this threshold. For samples larger than 13.85 μm, phonon-boundary scattering becomes negligible, *k*_*l*_ plateaus, and the bulk value of 2.35 W·m^-1^·K^-1^ is reached. Combined with the phonon dispersion shown in Fig 3, Ti and S atoms dominate the low- to mid-frequency regions, corresponding to the acoustic branches. Between 0 and 7.5 THz and between 8.1 and 12.1 THz, lattice vibrations are almost entirely contributed by Ti and S atoms. The significant overlap and interference of Ti- and S-related vibrations within these frequency windows serve as the primary drivers of lattice dynamics. Such interactions intensify phonon–phonon scattering and facilitate coupling between low-lying optical and acoustic branches, thereby reducing lattice thermal conductivity. A cluster of flat optical modes near 12.9–17.2 THz produces distinct peaks in the phonon density of states, arising almost entirely from boron vibrations. Phonon band gaps of 0.5 THz and 0.8 THz, respectively, separate the low- and mid-frequency optical branches, as well as the mid- and high-frequency optical branches.

**Fig 16:**
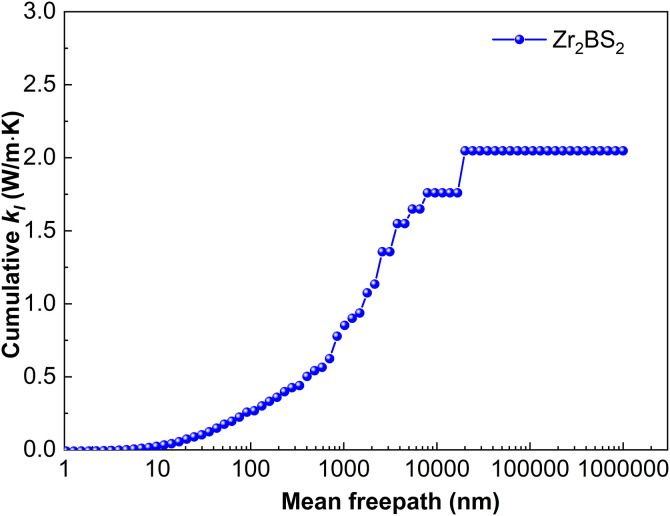
Cumulative lattice thermal conductivity of Zr_2_BS_2._

Fig 16 shows the cumulative lattice thermal conductivity of Zr_2_BS_2_ at 300 K as a function of MFP. Sample size is a key determinant: *k*_*l*_ stops increasing once the MFP exceeds 20,092.3 nm, indicating that all heat-carrying phonons have MFPs below this threshold. For samples larger than approximately 20.1 μm, boundary scattering becomes negligible, *k*_*l*_ plateaus, and the bulk value of 2.05 W·m^-1^·K^-1^ is reached. In conjunction with Fig 4, the vibrational frequencies associated with Zr and S atoms are located in the lowest-frequency region and correspond to the acoustic branches. Between 0 and 6.1 THz, lattice vibrations are dominated by Zr atoms, although the motions of Zr and S strongly overlap and jointly govern the dynamics within this range. Near 6.7 to 10.3 THz, the phonon branches flatten; Zr still contributes appreciably, but S atoms become the primary drivers of lattice vibrations in this frequency range. Zr–S vibrational interference within the 0–6.1 THz and 6.7–10.3 THz frequency ranges enhances scattering and facilitates coupling between low-energy optical and acoustic phonons. This interaction intensifies phonon–phonon interactions and suppresses lattice thermal conductivity. The 11.6–15.8 THz region is characterized by flat optical branches with prominent phonon density of states (PhDOS) peaks, which originate almost exclusively from B atoms. Phonon band gaps of 0.6 THz and 1.3 THz separate the low- and mid-frequency, and mid- and high-frequency optical branches, respectively.

**Fig 17:**
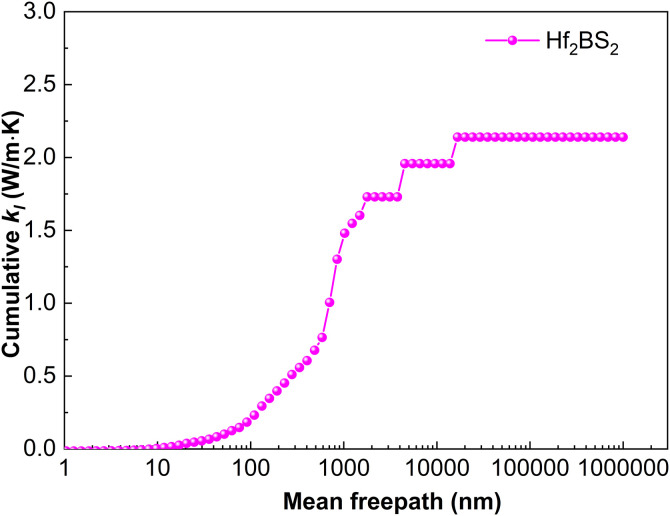
Cumulative lattice thermal conductivity of Hf_2_BS_2._

Fig 17 shows the cumulative lattice thermal conductivity of Hf_2_BS_2_ at 300 K as a function of the phonon mean free path. Sample size is a critical factor: *k*_*l*_ ceases to increase once the MFP exceeds 16,681.0 nm, indicating that all heat-carrying phonons have MFPs below this threshold. For samples larger than approximately 16.7 µm, boundary scattering becomes negligible, *k*_*l*_ saturates, and the bulk lattice thermal conductivity stabilizes at 2.14 W·m^-1^·K^-1^. In conjunction with Fig 5 the vibrational frequencies of Hf_2_BS_2_ are located in the lowest-frequency region, corresponding to the acoustic branches, and are predominantly influenced by Hf atoms, with a minor contribution from S atoms. In Hf₂BS₂, Hf atoms dominate lattice vibrations between 0–4.7 THz, while S atoms control the 6.8–9.8 THz range. Flat optical branches appear near 12.4–16.6 THz, yielding prominent phonon density of states (PhDOS) peaks that originate almost exclusively from boron vibrations. Phonon band gaps of 2.1 THz and 2.6 THz respectively separate low-/mid-frequency and mid-/high-frequency optical branches, stemming from the substantial mass differences among hafnium, sulfur, and boron.

Although the M_2_BS_2_ (M = Ti, Zr, Hf) compounds share nearly identical crystal structures and the mass difference among Ti, Zr, and Hf, their lattice thermal conductivities differ only slightly. Fig 3–Fig 5 reveal a common origin: the same crystal symmetry, a consistent phonon-scattering mechanism, and similar vibrational characteristics of the key atoms. Firstly, all three metals belong to group IVB, and their bonds with B and S exhibit comparable mixed ionic–covalent character and bond strength. Consequently, the interatomic force constants—which govern phonon dispersion and scattering strength—vary little among the three compounds, resulting in the observed minor variation in *k*_*l*_. Secondly, the dominant contribution to *k*_*l*_ in all M_2_BS_2_ compounds arises from low- and mid-frequency acoustic phonons (low energy, long mean free path). These phonon branches exhibit strong and comparably intense scattering in each material, effectively suppressing thermal conduction and minimizing differences among the three compounds. Thirdly, all three systems display pronounced coupling between low-lying optical and acoustic phonons, which further enhances phonon–phonon scattering and thereby inhibits heat transport. Because the energy overlap and coupling strength between the optical and acoustic branches are determined by the crystal structure and atomic vibrational patterns—which are nearly identical across the series—the suppressive effect of this coupling on *k*_*l*_ is similarly consistent in all M_2_BS_2_ phases. Moreover, the light mass and highly localized vibrations of the B atoms confine their high-frequency modes to a narrow region, which contributes negligibly to overall heat transport. This contribution is nearly identical across all three compounds. The mass difference among the metal atoms, therefore, does not alter the dominant scattering mechanisms in the low- and mid-frequency acoustic branches, so the lattice thermal conductivity *k*_*l*_ remains similar in value (2.05–2.35 W·m^-1^·K^-1^) for Ti_2_BS_2_, Zr_2_BS_2_, and Hf_2_BS_2_.

### 3.3. Electronic transport properties

#### 3.3.1. Electronic band structure.

**Fig 18:**
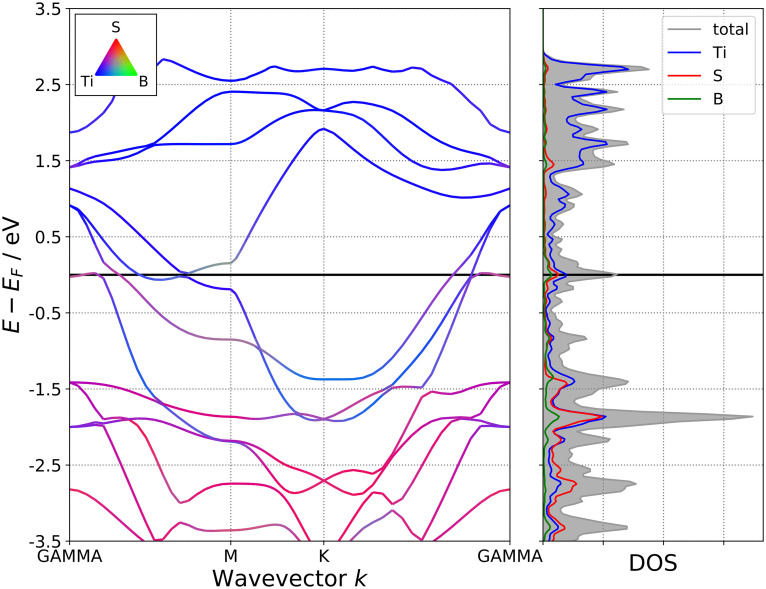
Electronic Band Structure of Ti_2_BS_2._

As two-dimensional MBene derivatives, the M_2_BS_2_ (M = Ti, Zr, Hf) sheets exhibit metallic behavior, with the conduction band crossing the Fermi level (*E*_*f*_). This is evidenced by the band structures shown in Fig 18–Fig 20, where the energy bands intersect *E*_*f*_ along the high-symmetry k-path. This metallicity arises from the strong delocalization of the transition-metal d electrons, which hybridize with the p orbitals of interstitial boron and surface sulfur atoms, producing a continuous density of states at *E*_*f*_ without any band gap. The transition metal M is the primary contributor to the conduction band, as its high-energy d orbitals lie closest to the Fermi level. Both boron and sulfur also contribute significantly near *E*_*f*_, while their 2p orbitals predominantly form the valence band. Through M–B and M–S bonding, these atoms influence the overall electron distribution throughout the lattice.

**Fig 19:**
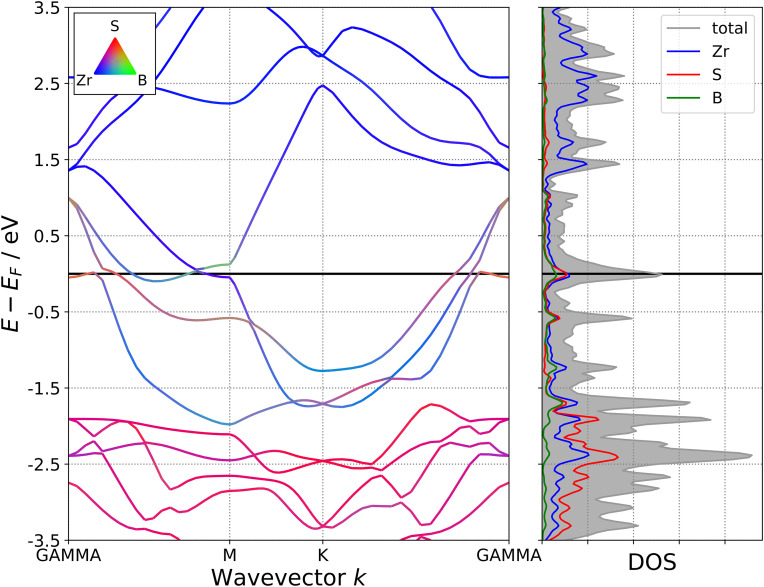
Electronic Band Structure of Zr_2_BS_2._

**Fig 20:**
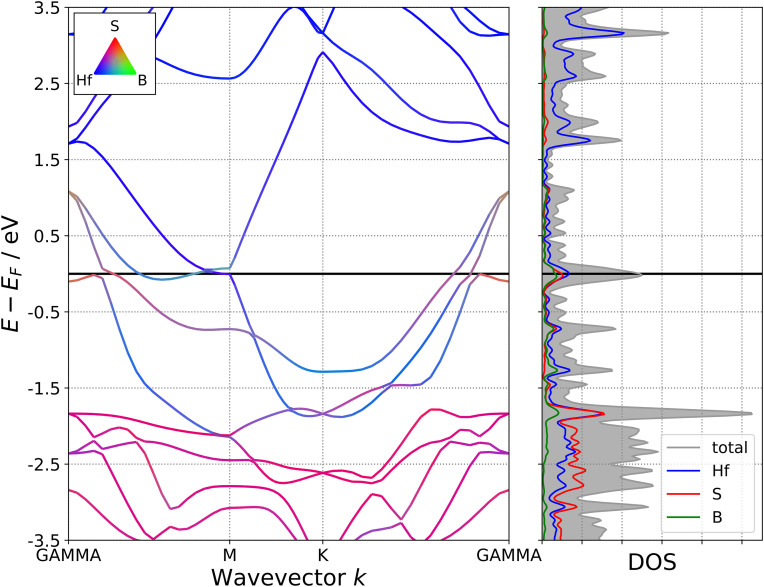
Electronic Band Structure of Hf_2_BS_2._

#### 3.3.2. Electrical conductivity, electronic thermal conductivity, Seebeck coefficient, power factor and thermoelectric merit.

**Fig 21:**
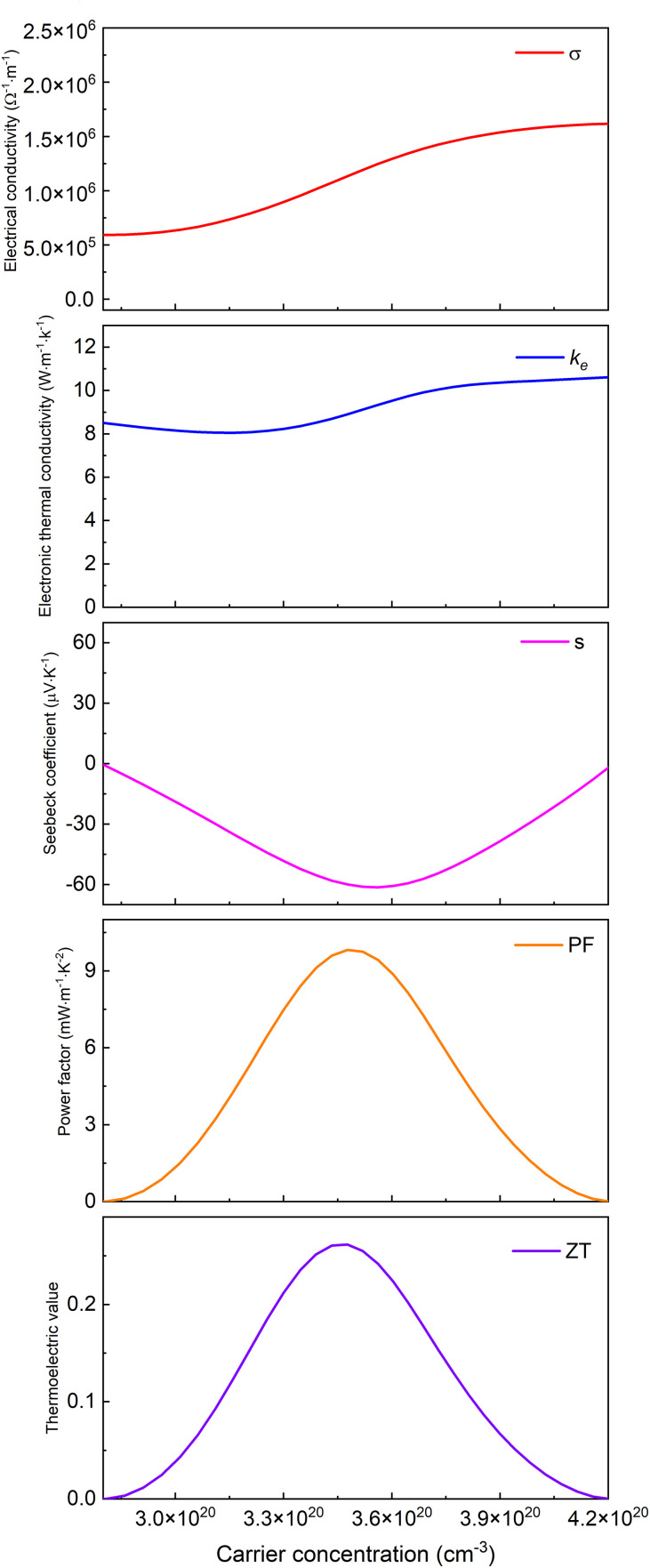
Seebeck coefficient, *σ*, *PF*, *κ*_*e*_ and *zT* of p-doped of Ti_2_BS_2._

Based on Boltzmann transport theory within the constant relaxation time approximation, the electronic transport properties of monolayer M_2_BS_2_ were calculated using the BoltzTraP code. Fig 21–Fig 26 show the dependence of the relevant transport coefficients on doping concentration at 300 K. Specifically, Fig 21–Fig 23 correspond to p-type doping and present the electrical conductivity (*σ*), electronic thermal conductivity (*k*_*e*_), Seebeck coefficient (*S*), power factor (*PF* = *S²σ*), and thermoelectric figure of merit (*zT*). Similarly, Fig 24–Fig 26 display the same quantities for n-type doping. These quantities are evaluated using the following expressions:

**Fig 22:**
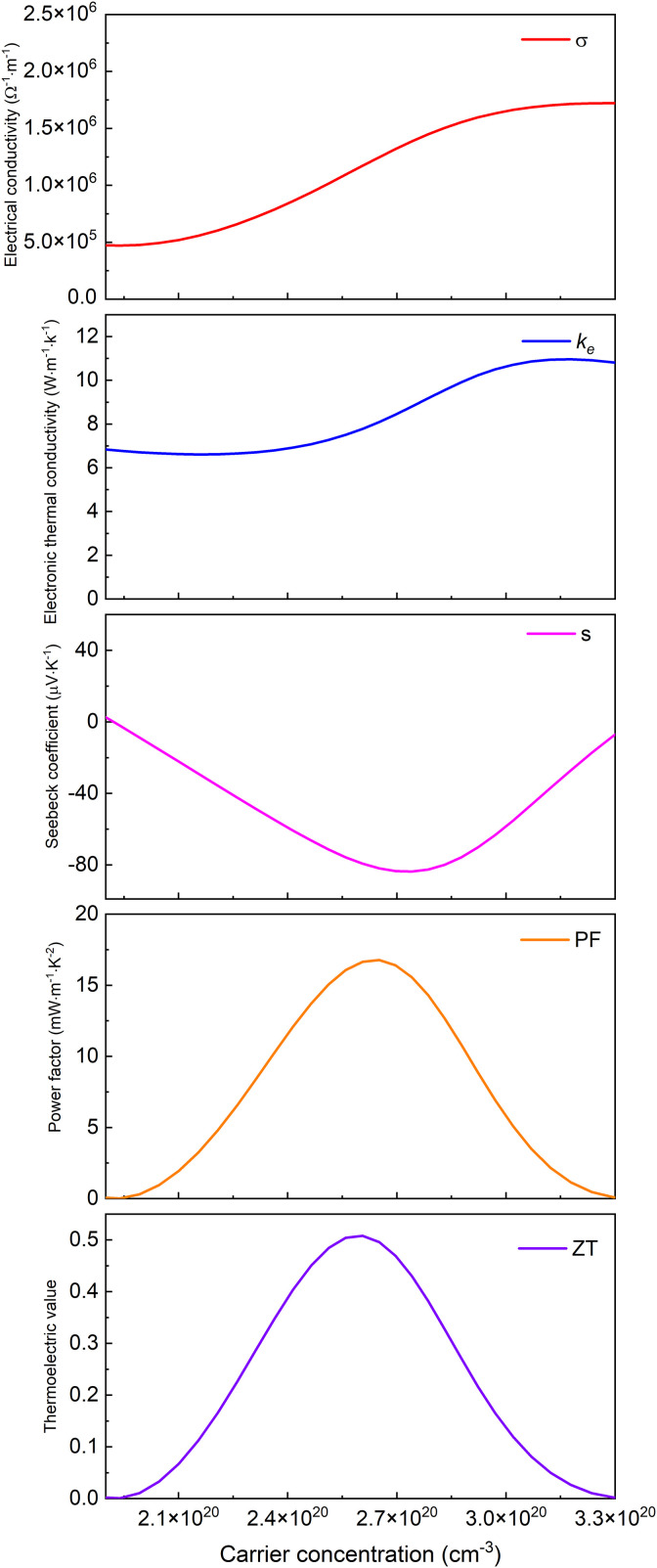
Seebeck coefficient, *σ*, *PF*, *κ*_*e*_ and *zT* of p-doped of Zr_2_BS_2._

**Fig 23:**
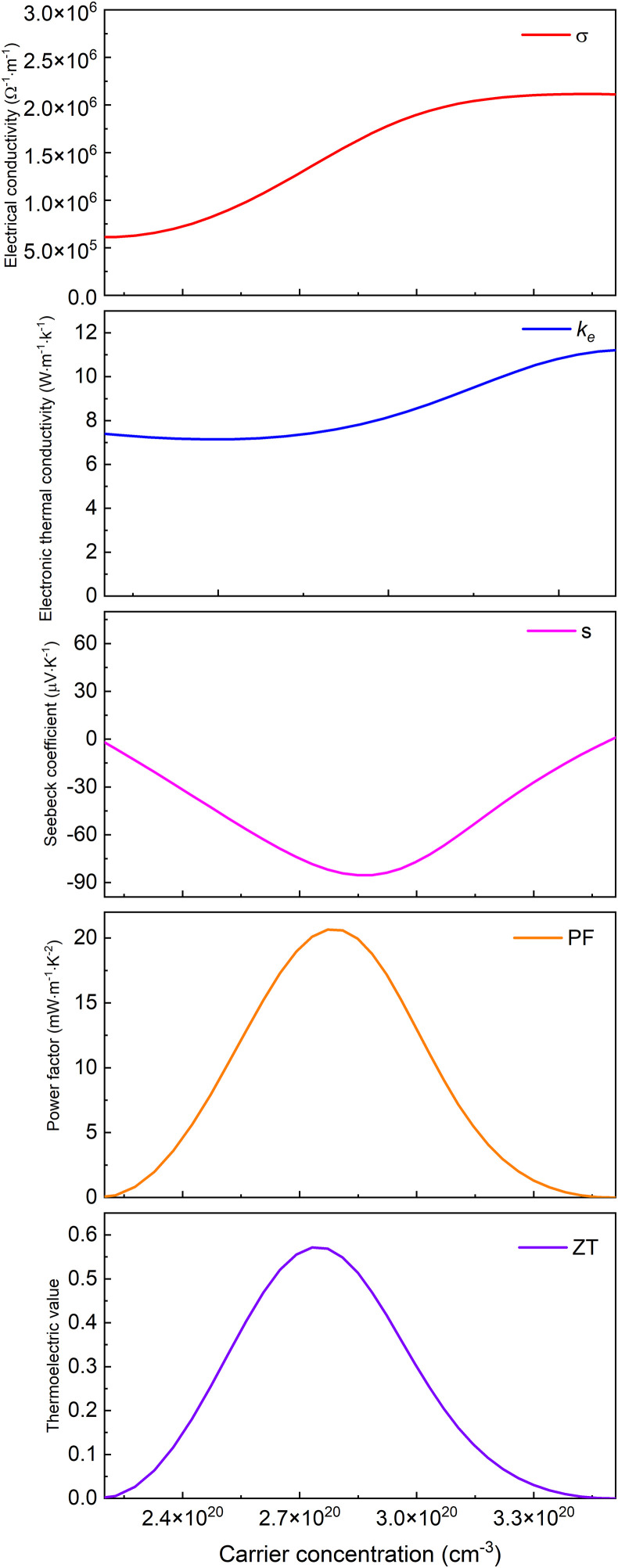
Seebeck coefficient, *σ*, *PF*, *κ*_*e*_ and *zT* of p-doped of Hf_2_BS_2._

**Fig 24:**
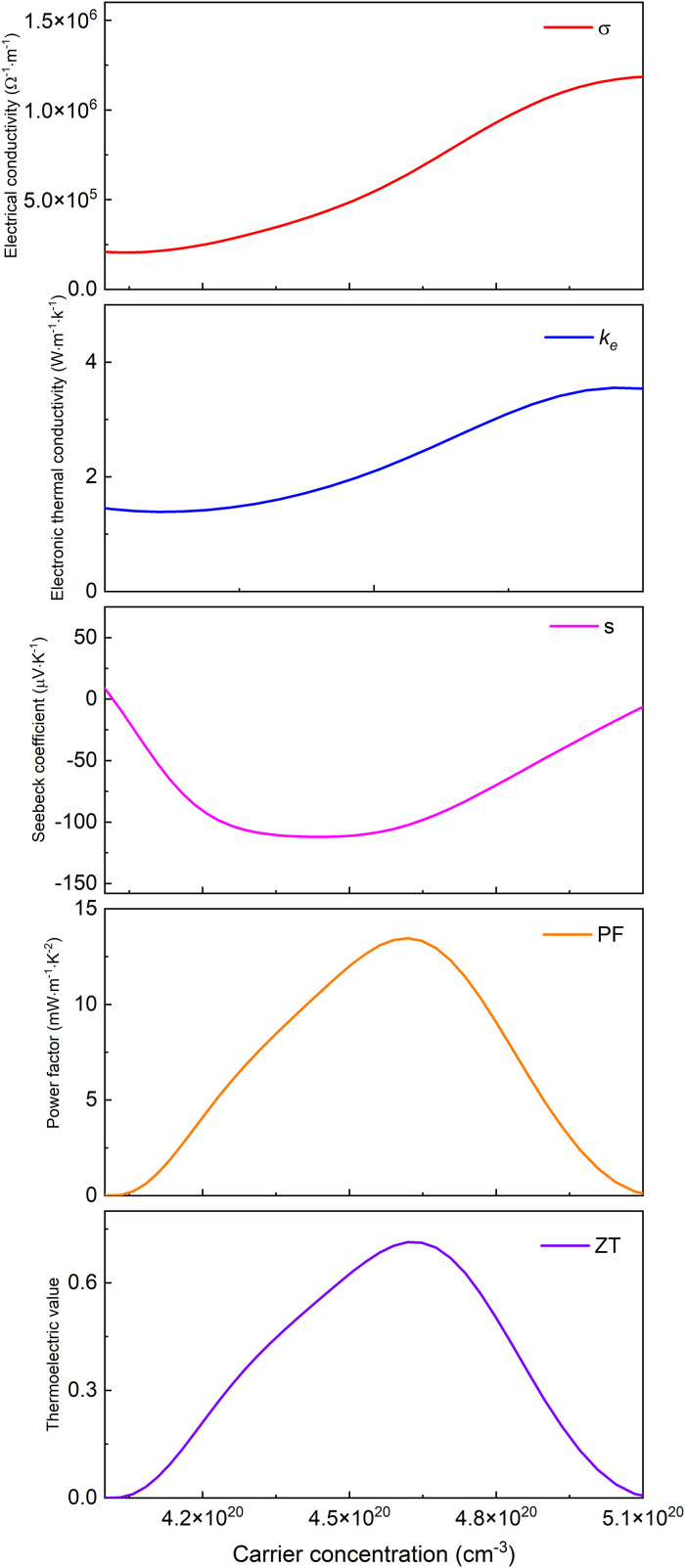
Seebeck coefficient, *σ*, *PF*, *κ*_*e*_ and *zT* of n-doped of Ti_2_BS_2._

**Fig 25:**
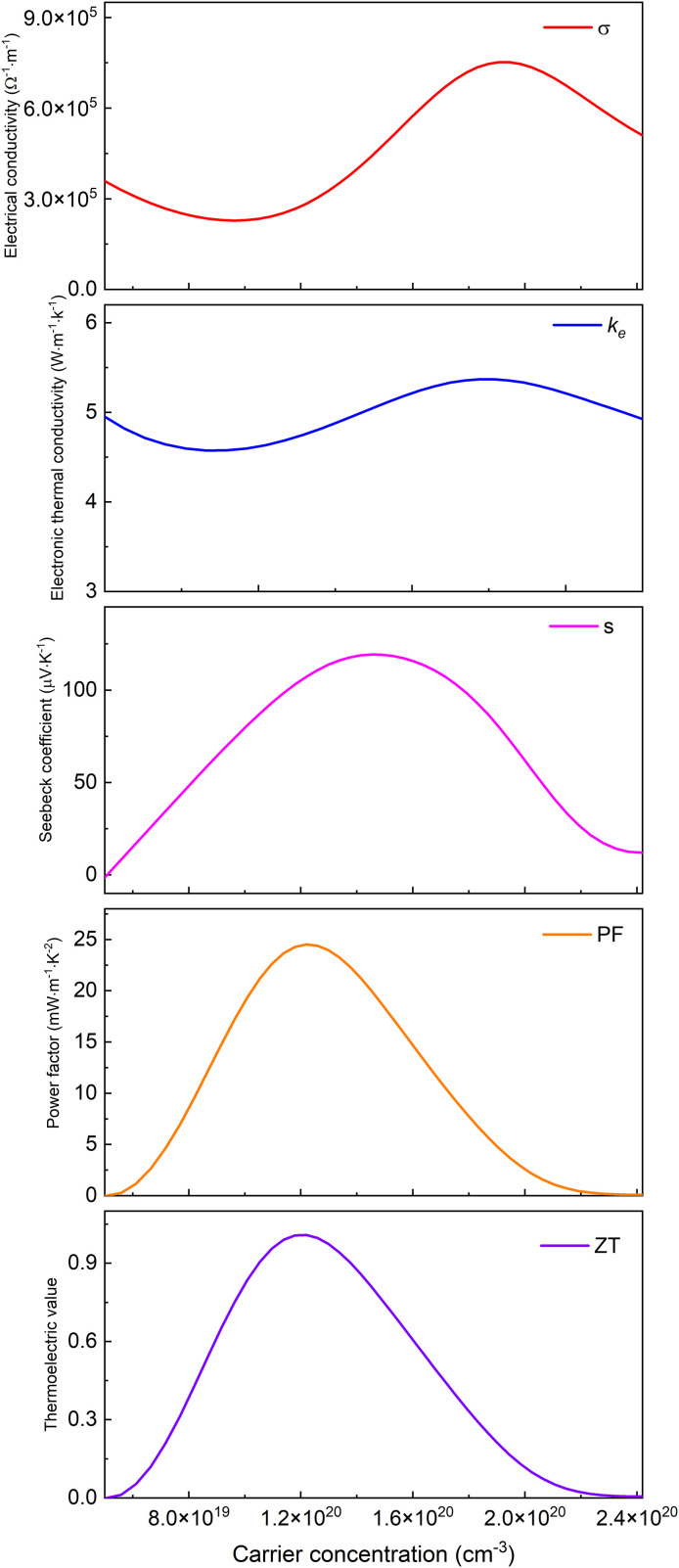
Seebeck coefficient, *σ*, *PF*, *κ*_*e*_ and *zT* of n-doped of Zr_2_BS_2._

**Fig 26:**
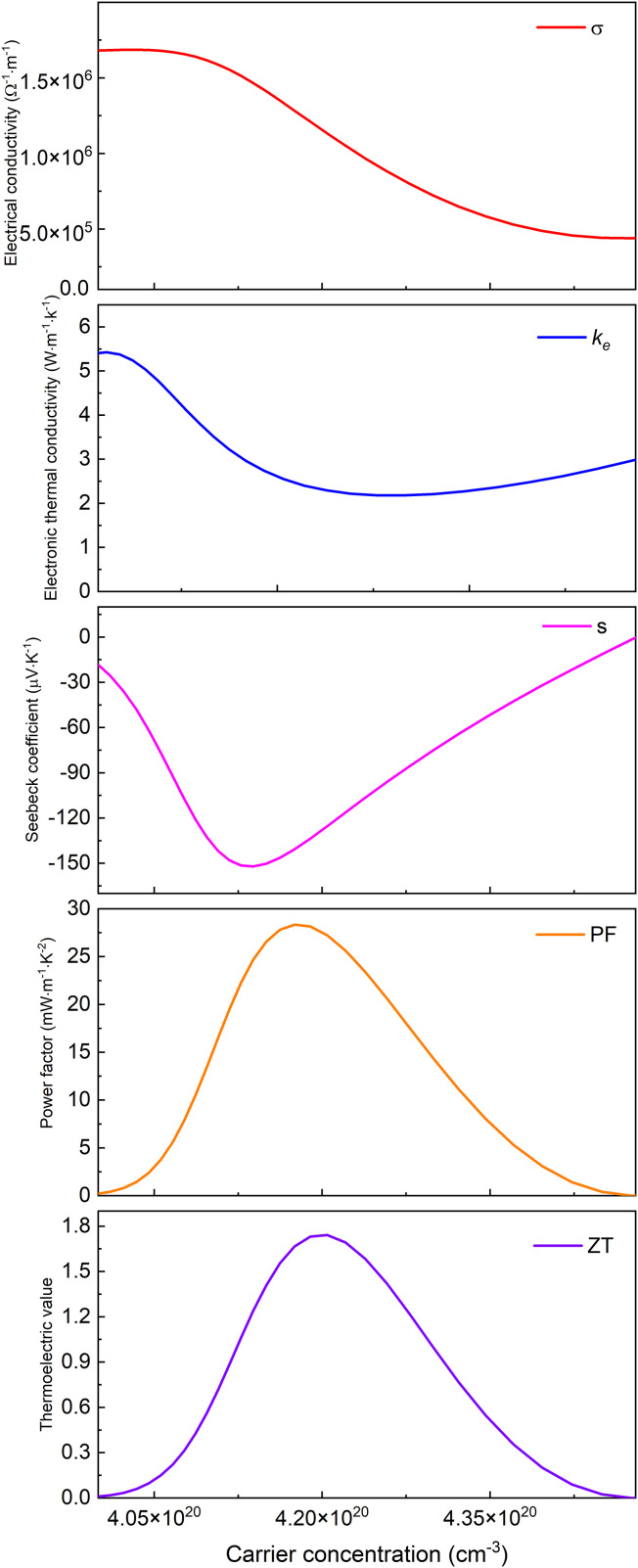
Seebeck coefficient, *σ*, *PF*, *κ*_*e*_ and *zT* of n-doped of Hf_2_BS_2._


$$S_{\alpha \beta }(T,\mu )=\frac{\text{1}}{eTV\sigma_{\alpha \beta }(T,\mu )}\times \int \sum_{\alpha \beta }(\varepsilon )(\varepsilon -\mu )\left[-\frac{\partial f_{\mu }(T,\mu )}{\partial \varepsilon }\right]\text{d}\varepsilon$$ (2)

$$\sigma_{\alpha \beta }(T,\mu )=\frac{\text{1}}{V}\int \sum_{\alpha \beta }(\varepsilon )\left[-\frac{\partial f_{\mu }(T,\mu )}{\partial \varepsilon }\right]\text{d}\varepsilon$$ (3)

$$\sum_{\alpha \beta }(\varepsilon )=\frac{e^{\text{2}}}{N_{\text{0}}}\sum_{i,k}\tau v_{\alpha }(i,k)v_{\beta }(i,kfrac\delta \left(\varepsilon -\varepsilon_{i,k}\right)\text{d}\varepsilon$$ (4)

$$PF={\sigma S}^{\text{2}}$$ (5)

$$ZT=\frac{{\sigma S}^{\text{2}}}{k_{l}+k_{e}}$$ (6)

Among these, V is the volume of the unit cell, $\;\sum_{\alpha \beta }(\varepsilon )$ is the transport distribution function, and $f_{\mu }(T,\mu )$ is the Fermi–Dirac distribution function. *τ* is the relaxation time, *v* is the carrier velocity, *D*_*(ε)*_ is the density of states, *f* is the Fermi–Dirac distribution, *μ* is the chemical potential, and *k*_*l*_ is the lattice thermal conductivity.

Fig 21–Fig 23 show the dependence of the Seebeck coefficient, electrical conductivity, power factor, electronic thermal conductivity, and thermoelectric figure of merit in the p-type doping concentration for monolayer M_2_BS_2_. Within the doping range where *zT* peaks, the electrical conductivity of all three materials decreases monotonically with increasing hole concentration. The conductivities are ordered as *σ*(Hf_2_BS_2_)> *σ*(Zr_2_BS_2_)> *σ*(Ti_2_BS_2_), with maximum values of approximately 2.17 × 10^6^ Ω^-1^·m^-1^, 1.70 × 10^6^ Ω^-1^·m^-1^, and 1.52 × 10^6^ Ω^-1^·m^-1^, respectively.

Within the p-type doping window that maximizes the figure of merit, the electronic thermal conductivities follow the trend *k*_*e*_(Hf_2_BS_2_) > *k*_*e*_(Zr_2_BS_2_) > *k*_*e*_(Ti_2_BS_2_), with peak values of approximately 11.23W·m^-1^·K^-1^, 10.83W·m^-1^·K^-1^, and 10.62 W·m^-1^·K^-1^, respectively. The corresponding Seebeck coefficients differ only modestly. Their extrema decrease in magnitude as |*S*|(Hf_2_BS_2_)>|*S*|(Zr_2_BS_2_)> |*S*|(Ti_2_BS_2_), reaching −85.3 μV·K^-1^, −83.6μV·K^-1^, and −61.3μV·K^-1^, respectively, with Hf_2_BS_2_ exhibiting the largest |*S*|.

Within the p-type doping windows that maximize their respective *zT* values, the monolayers achieve peak power factors of 9.8 mW·m^-1^·K^-2^, 16.8 mW·m^-1^·K^-2^, and 20.7 mW·m^-1^·K^-2^ for Ti_2_BS_2_, Zr_2_BS_2_, and Hf_2_BS_2_, respectively, with Hf_2_BS_2_ exhibiting the highest power factor. Using these optimized electronic transport coefficients—Seebeck coefficient (*S*), electrical conductivity (*σ*), and electronic thermal conductivity *k*_*e*_—and substituting them into the thermoelectric figure of merit expression (Eq. 6), we obtain maximum *zT* values of 0.26, 0.51, and 0.57 for Ti_2_BS_2_, Zr_2_BS_2_, and Hf_2_BS_2_ under p-type doping.

Fig 24–Fig 26 illustrate the n-type doping dependence of the Seebeck coefficient, electrical conductivity, power factor, electronic thermal conductivity, and thermoelectric figure of merit for Ti_2_BS_2_, Zr_2_BS_2_, and Hf_2_BS_2_. Within the doping ranges that maximize *zT*, the electrical conductivity of Ti_2_BS_2_ and Hf_2_BS_2_ increases with rising electron concentration, whereas that of Zr_2_BS_2_ decreases. The peak conductivities follow the order σ(Hf_2_BS_2_)> σ(Ti_2_BS_2_)> σ(Zr_2_BS_2_), with maximum values of approximately 1.69 × 10^6^ Ω^-1^·m^-1^, 1.18 × 10^6^ Ω^-1^·m^-1^, and 7.52 × 10^5^ Ω^-1^·m^-1^, respectively.

Under n-type doping and within the concentration ranges that maximize their individual ZT values, the electronic thermal conductivities follow the trend *k*_*e*_(Hf_2_BS_2_) > *k*_*e*_(Zr_2_BS_2_) > *k*_*e*_(Ti_2_BS_2_), with peak values of 5.43, 5.39, and 3.69 W·m^-1^·K^-1^, respectively. The corresponding Seebeck coefficients decrease in magnitude as |*S*|(Hf_2_BS_2_) <|*S*|(Zr_2_BS_2_) <|*S*|(Ti_2_BS_2_), with extrema of −152.1μV·K^-1^, −119.3μV·K^-1^, and −112.0 μV·K^-1^ for Ti_2_BS_2_, Zr_2_BS_2_, and Hf_2_BS_2_, respectively.

Ti_2_BS_2_ and Zr_2_BS_2_ exhibit Seebeck coefficient maxima that are nearly identical in magnitude, whereas Hf_2_BS_2_ has the largest Seebeck coefficient maximum. Concurrently, the thermoelectric power factors of the three materials decrease in the order *PF*(Hf_2_BS_2_)> *PF*(Zr_2_BS_2_)> *PF*(Ti_2_BS_2_). Within the doping concentration range that yields the peak thermoelectric figure of merit, their maxima are 13.5 mW·m^-1^·K^-2^, 24.5 mW·m^-1^·K^-2^, and 28.4 mW·m^-1^·K^-2^, respectively.

Based on the results, Hf_2_BS_2_ exhibits a superior thermoelectric power factor compared to the other two materials. Under n-type doping, the peak thermoelectric figure of merit (*zT*_*max*_) for monolayer Ti_2_BS_2_, Zr_2_BS_2_, and Hf_2_BS_2_ is 0.71, 1.01, and 1.74, respectively. Fig 21–Fig 26 show that, across the entire doping concentration range, the thermoelectric performance of all three materials is significantly higher under n-type doping than under p-type doping. To date, experimental synthesis of M_2_BS_2_ (including Hf_2_BS_2_) monolayers remains unreported, and these materials exist as theoretical predictions. Their experimental realization would likely require chemical vapor deposition or molecular beam epitaxy approaches, with high-quality defined as >100 μm single-crystal domains and carrier mobility >100 cm^2^·V^-1^·s^-1^ [52–59].

In summary, the two-dimensional Ti_2_BS_2_, Zr_2_BS_2_, and Hf_2_BS_2_ monolayers share similar crystal structures; however, their thermoelectric figures of merit differ significantly. Among them, Hf_2_BS_2_ exhibits the highest *zT* under n-type doping. At room temperature, the lattice thermal conductivities are 2.35 W·m^-1^·K^-1^ for Ti_2_BS_2_, 2.05 W·m^-1^·K^-1^ for Zr_2_BS_2_, and 2.14 W·m^-1^·K^-1^ for Hf_2_BS_2_. The low lattice thermal conductivities of all three materials are closely linked to their pronounced anharmonicity and large Grüneisen parameters. The combination of a substantial Seebeck coefficient and a high thermoelectric power factor in Hf_2_BS_2_ is the primary factor contributing to its superior thermoelectric figure of merit. These findings provide valuable insights for the development of novel, high-performance thermoelectric device materials.

## 4. Conclusion

This study investigates the thermoelectric properties of two-dimensional M_2_BS_2_ materials. The results reveal that these materials adopt a layered trigonal structure characterized by strong in-plane covalent bonding and van der Waals interlayer interactions. The lattice parameters scale with the radius of the M atom, and all phases are dynamically stable. Regarding phonon transport, the lattice thermal conductivity is predominantly governed by acoustic and low-frequency optical phonons, following the sequence Ti_2_BS_2_ (2.35 W·m^-1^·K^-1^)> Hf_2_BS_2_ (2.14 W·m^-1^·K^-1^)> Zr_2_BS_2_ (2.05 W·m^-1^·K^-1^). Zr_2_BS_2_ exhibits the lowest thermal conductivity due to its pronounced anharmonicity and high phonon scattering rate. Additionally, under n-type doping, the *zT* of M_2_BS_2_ is significantly higher than under p-type doping, with Hf_2_BS_2_ achieving the highest *zT* of 1.74 due to its superior Seebeck coefficient and power factor. This study confirms that the M_2_BS_2_ family is a highly promising class of two-dimensional thermoelectric materials and provides a solid theoretical foundation for future experimental synthesis and device design.
